# Real-world outcomes of Defocus Incorporated Multiple Segments lenses on retarding axial elongation in myopic children and adolescents

**DOI:** 10.3389/fmed.2024.1416286

**Published:** 2025-01-24

**Authors:** Rachel K. M. Chun, Kryshell Y. Q. Wong, Carly S. Y. Lam, Chi-ho To, Kenneth K. K. Liu, Yin-zhi Wong, Wing-chun Tang, Nicole Chan, Dora Kwok, Max Cheung, David Yung, Andrew K. C. Lam

**Affiliations:** ^1^School of Optometry, The Hong Kong Polytechnic University, Kowloon, Hong Kong SAR, China; ^2^Centre for Eye and Vision Research (CEVR), Hong Kong, Hong Kong SAR, China; ^3^Research Centre for SHARP Vision (RCSV), The Hong Kong Polytechnic University, Kowloon, Hong Kong SAR, China; ^4^School of Optometry & Vision Sciences, Cardiff University, Cardiff, United Kingdom

**Keywords:** myopia, axial elongation, defocus, DIMS, myopic control, real-world outcomes

## Abstract

**Purpose:**

This study aimed to examine the effect of Defocus Incorporated Multiple Segments (DIMS) lenses on myopia progression and axial elongation in a clinical population.

**Methods:**

A retrospective study was conducted using clinical data from 489 and 156 patients aged 3 to 17 years old who were prescribed DIMS and single vision (SV) lenses, respectively at the Optometry Clinic of The Hong Kong Polytechnic University between July 2018 and August 2019. The study included patients with previous myopia control interventions. The changes in spherical equivalent refraction (SER) and axial length (AL) were measured and normalized to annual changes. The correlation between age at baseline and annual change in AL was also examined.

**Results:**

The total change in SER and AL after DIMS were −0.94 ± 0.79D and 0.55 ± 0.40 mm, respectively with an average wearing period of 31.98 ± 9.97 months. The normalized annual changes in SER and AL in DIMS wearers were significantly smaller than those in SV wearers (DIMS; SER change vs. AL changes; −0.38 ± 0.32D vs. 0.22 ± 0.16 mm. SV; −0.45 ± 0.41D vs. 0.29 ± 0.20 mm, *p* < 0.05). Patients with a history of myopia control had greater myopia progression after wearing DIMS lenses. There was a significant negative correlation between age at baseline and annual change in AL (correlation coefficient, *r* = −0.61, *p* < 0.001), suggesting that myopia progression was faster in children with a younger age of onset. A small proportion of patients (2.7%) experienced a clinically significant axial shortening (total change in AL:−0.13 ± 0.07 mm) after wearing DIMS lenses more than 2 years.

**Conclusion:**

The study demonstrated that DIMS lenses could retard axial elongation, with the effect sustained with increased duration of lens wear. However, patients with previous myopia control experienced greater myopia progression after wearing DIMS lenses. The study also highlighted the potential for axial length shortening in a small proportion of patients after the DIMS lens wear. These findings underscore the importance of adherence to intervention in achieving optimal treatment efficacy. Further research is needed to understand the mechanisms underlying these effects and to optimize the use of optical interventions in myopia control.

## 1 Introduction

The prevalence of myopia has been increasing globally especially in East Asian areas including China, Hong Kong, Taiwan, and Singapore (1). It is projected that by 2050, approximately half of the world’s population will have myopia (2). The rapid rise of high myopia, particularly at younger ages, suggests a longer duration of myopia progression prior to stabilization in adulthood, leading to a higher incidence of high myopia in the future. Complications of high myopia such as retinal detachment and degenerations are mainly due to excessive stretching of eyeball. Studies on myopia including interventional studies should therefore include the information of axial length (3, 4).

Optical and pharmaceutical approaches have demonstrated efficacy in reducing myopia progression. Optical interventions include orthokeratology, soft contact lenses such as multifocal, dual focus, and defocus-incorporated lenses, as well as specially-designed spectacle lenses that produce myopic defocus through multiple segments or aspherical lenslets, or modify contrast (5, 6).

Spectacle wear is the easiest and widely accessible method for myopia control. Defocused incorporated multiple segments (DIMS) which incorporates multiple + 3.5D myopic defocus segments at peripheral field. A double masked randomized clinical trial has demonstrated 52% slower in myopia progression and 62% less axial elongation after wearing DIMS lens compared with those wearing single vision lens over two years (7). The myopia control effect could be sustained in the third year (8). Apparently, there was no rebound effect after discontinuing DIMS lens wear. Since the launch of DIMS lens in 2018 (9), Long et al. (10) had 1-year retrospective study comparing DIMS lens wearers to single vision (SV) lens wearers. Liu et al. (10) published the first retrospective real-world study comparing DIMS lens wearers vs. single vision (SV) lens wearers for two years. The main limitation of these retrospective studies lacked important information such as axial length measurement, best corrected visual acuity (BCVA), and binocularity (10, 11). Although Liu et al. (11) considered their study adopted real world research design, they excluded subjects who had applied other interventions for myopia control. There was no mention about best corrected visual acuity (BCVA) or binocularity of their subjects. The efficacy of DIMS lenses in children with a history of myopia control, concurrent myopia control treatment, BCVA less than 0.00 logMAR, or strabismus remains a topic of uncertainty for many eyecare professionals.

While randomized controlled trials are considered as the gold standard for generating clinical evidence, real-world data provide valuable insight to evaluate efficacy of interventions in actual clinical settings. Randomized controlled trials are conducted with strict inclusion and exclusion criteria. In real-world study, patients with comorbidities and concurrent therapies should not be excluded. In real clinical practice, patients will be treated as long as the risk to benefit ratio appears favorable (12). Real-world data can also inform design of future randomized clinical trials (13).

In view of the above considerations, we sought to extract the clinical data including axial length measurement, from the university-based clinic to examine the effect of DIMS lenses on myopia progression in clinic population.

## 2 Materials and methods

This was a retrospective, observational cohort study. The study was conducted in accordance with the tenets of the Declaration of Helsinki, and approval was obtained from the Institutional Review Board of The Hong Kong Polytechnic University (HSEARS20220222004). This retrospective study adopted the clinical data from the Optometry Clinic within the university campus. Electronic patient records were retrieved in summer 2022 for patients who prescribed DIMS or single vision (SV) spectacle lenses during July 2018 to August 2019. We included Chinese patients who had both subjective refraction and axial length results in the visit that DIMS or SV spectacle lenses were first prescribed (baseline) and the latest visit (final) by summer 2022. The wearing duration is defined as the number of months that patients had been using DIMS or SV lenses as of the data retrieval period in the summer of 2022. Their wearing duration of DIMS or SV spectacle lenses had to be at least 11.5 months. DIMS or SV spectacle lenses could be replaced in regular follow-up visits when a change of spherical equivalent refraction was more than 0.50D (7). Patients with tropia and anisometropia equal or more than 1.75D were excluded from the data analysis.

Other patient information such as age at the baseline visit, gender, best-corrected visual acuity (BCVA) and binocularity were extracted. Axial length could be measured by an optical biometer either using IOL Master 500 (Carl Zeiss Meditec, AG) or AL-Scan (Nidek, Japan). Keratometry results were extracted if available. Cycloplegic refraction by either one drop of 1% cyclopentolate or two drops of 1% tropicamide in 5-min interval was not an inclusion criterion. Patients with previous myopia control interventions were also included.

### 2.1 Statistical analysis

Raw data was tested for normality using the Kolomogorov-Smirnov test. The correlation between two eyes in term of spherical equivalent refraction (SER) and axial length (AL) were examined using Spearman’s correlation. Myopia, in terms of spherical equivalent refraction (SER) and axial length (AL) of the two eyes were highly correlated both at baseline (SER: Ro = 0.849, *p* < 0.001; AL: *R* = 0.937, *p* < 0.001) and in the final visit (SER: Ro = 0.807, *p* < 0.001; AL: *R* = 0.888, *p* < 0.001). Only results of the right eye were analyzed and presented. Total change of myopia (∆SER) and change of axial length (∆AL) over the wearing period was calculated. Annual changes in SER and AL were then normalized to the wearing duration individually (Annual changes in SER or AL = (Total changes/total wearing duration in months) × 12 months).

**Figure 1:**
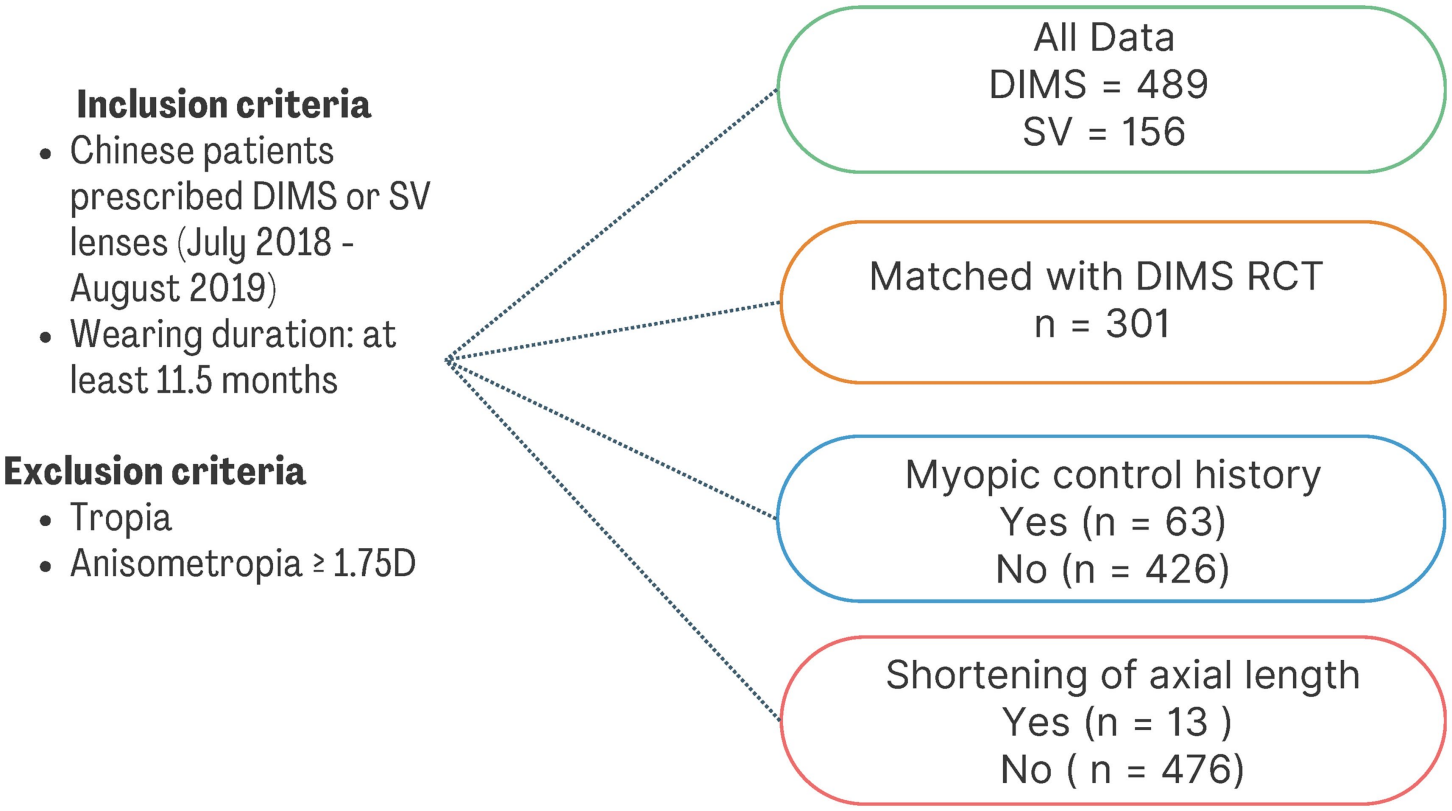
A flow chart illustrating the different grouping of data analysis. DIMS, Defocus Incorporated Multiple Segments; SV, Single Vision; RCT, randomized controlled study.

The relationship of ∆SER or ∆AL and baseline age, SER and wearing duration was explored using Spearman’s correlation. Data were expressed as mean ± standard deviation (SD). All data analyses were performed using SPSS software 29.0.2.0 (IBM Corporation). Figure 1 showed a flow chart illustrating the different grouping of data analysis.

## 3 Results

The distribution of AL at baseline was normal (*p* > 0.05, Kolomogorov-Smirnov test) while the age, spherical power, cylindrical power and SER at baseline violated the assumption of normality. In this retrospective study, 489 patients who prescribed DIMS lenses and 156 patients who prescribed SV lenses for at least 11.5 months were included. Only right eyes were selected for data analysis.

### 3.1 Baseline demographics

The age range of the patients who prescribed DIMS lenses was from 3 to 17 years old, with mean age of 8.75 ± 2.06 years. Fifty percent of the patients (248 out of 489) received cycloplegic refraction. Their mean magnitude of myopia and astigmatism at baseline was −2.66 ± 1.31D and −0.73 ± 0.75D, respectively (Table 1). The average wearing period of DIMS lenses was 31.98 ± 9.97 months. Around 13 % of the patients (63 out of 489) had previous myopic control interventions or received combination interventions of low dose atropine. Among these 63 patients, 49 of them concurrently adopted DIMS lenses and low dose atropine as myopia control intervention. The remaining 14 patients with history of myopia control switched to wearing DIMS lenses.

**Table 1:** Baseline demographics and characteristics of all patients (DIMS wearer = 489 and SV wearer = 156) in the dataset.

Baseline	DIMS wearers		SV wearers		Mann–Whitney U test
	Mean ± SD	Range	Mean ± SD	Range	
Age (years)	8.75 ± 2.06	3 to 17	8.76 ± 3.23	3 to 16	*p* = 0.728
Male: female	248:241		86:70		*p* = 0.337
Cycloplegic refraction	Yes: No = 248:241		Yes: No = 62:94		*p* = 0.017
Spherical refraction (D)	−2.66 ± 1.31	−0.25 to −8.00	−1.79 ± 2.26	1.50 to −12.50	*p* < 0.001
Cylindrical refraction (D)	−0.73 ± 0.75	0.00 to −4.00	−1.14 ± 1.10	0.00 to −5.00	*p* < 0.001
Spherical equivalent refraction (D)	−3.03 ± 1.39	−0.50 to −8.38	−2.36 ± 2.22	−0.50 to −13.38	*p* < 0.001
Axial length (mm)	24.60 ± 0.93	22.40 to 28.04	24.20 ± 1.27	21.18 to 28.69	*p* < 0.001^a^
Anisometropia (D)	−0.02 ± 0.55	−1.50 to 1.50	−0.03 ± 0.55	−1.50 to 1.13	*p* = 0.815
Wearing period (months)	31.98 ± 9.97	11.50 to 50.50	29.05 ± 8.76	12.10 to 46.90	*p* < 0.001
Myopic control ^1^	Yes: No = 63:426		Yes: No = 0:156		*p* < 0.001
After wearing DIMS lenses			After wearing SV lenses		
Total ∆SER (D)	−0.94 ± 0.79	0.88 to −3.13	−1.01 ± 0.85	−3.50 to 2.38	*p* = 0.537
Total ∆AL (mm)	0.55 ± 0.40	−0.29 to 1.72	0.66 ± 0.46	−0.16 to 2.13	*p* = 0.077^b^
Normalized annual ∆SER (D)	−0.38 ± 0.32	0.29 to −1.63	−0.45 ± 0.41	−2.00 to 1.38	*p* = 0.030
Normalized annual ∆AL (mm)	0.22 ± 0.16	−0.13 to 0.78	0.29 ± 0.20	−0.05 to 1.01	*p* < 0.001^b^ ***

1: Number of patients who had previous myopic control interventions or receiving both DIMS lenses and low dose atropine a: Unpaired *t*-test b: One-way ANCOVA, ***p < 0.001 after controlling for axial length at baseline.

Those patients who prescribed SV lenses had a similar mean age (8.75 ± 3.23 years old). Their mean magnitude of spherical equivalent refraction at baseline was less myopic compared to those DIMS wearers (SV vs. DIMS; −2.36 ± 2.22D vs. −3.03 ± 1.39D, *p* < 0.001).

### 3.2 Effect of DIMS lenses on refraction and axial length

The total ∆SER and ∆AL after DIMS lenses were −0.94 ± 0.79D and 0.55 ± 0.40 mm, respectively. Since the current real-world analysis included different duration of DIMS lens wear, normalization to annual changes was required. The normalized annual ∆SER and ∆AL were −0.38 ± 0.32D and 0.22 ± 0.16 mm, respectively (Table 1). The total ∆SER and ∆AL after SV lenses were –1.01 ± 0.85D and 0.66 ± 0.46 mm, respectively with an average wearing period of 29.05 ± 8.76 months. When normalized to annual changes, the changes were −0.45 ± 0.41D and 0.29 ± 0.20 mm, respectively. A significant smaller annual axial elongation was found in DIMS wearer (*p* < 0.001).

To better compare to the previous randomized controlled trial (RCT) on DIMS lenses (7), 301 patients aged 8 to 13 years were selected from the current dataset. All of them had normal visual acuity, no history of myopia control and using DIMS lenses alone in the study period. They had no tropia or other binocular misalignment. The baseline demographics including gender distribution, SER (either with or without cycloplegia), axial length and average DIMS wearing period were tabulated (Table 2). The normalized annual ∆SER and ∆AL were significantly smaller in this data subset compared to the control group in our previous RCT (Annual ∆SER: current DIMS wearer vs. SV wearer in RCT; −0.29 ± 0.28D vs. −0.55 ± 0.36D, *p* < 0.001, unpaired *t*-test. Annual ∆AL: current DIMS wearer vs. SV wearer in RCT; 0.17 ± 0.13 mm vs. 0.32 ± 0.18 mm, *p* < 0.001, unpaired *t*-test).

**Table 2:** Baseline demographics and characteristics of 301 patients matched with previous randomized controlled trial of two-year DIMS lens wear (7).

Baseline	Mean ± SD	Range
Age (years)	9.61 ± 1.49	8 to 13
Male: female	154:147	
Cycloplegic refraction	Yes: No = 157:144	
Spherical refraction (D)	−2.787 ± 1.26	−0.25 to −6.75
Cylindrical refraction (D)	−0.75 ± 0.73	0.00 to −4.00
Spherical equivalent refraction (D)	−3.15 ± 1.36	−0.63 to −6.88
Axial length (mm)	24.78 ± 0.89	22.62 to 27.19
Anisometropia (D)	−0.04 ± 0.60	−1.50 to 1.50
Wearing period (months)	32.15 ± 9.82	11.60 to 48.10
Myopic control history	Yes: No = 0:301	
Presence of tropia	Yes: No = 0:301	
After wearing DIMS lenses		
Total ∆SER (D)	−0.74 ± 0.70	0.88 to −3.00
Total ∆AL (mm)	0.43 ± 0.33	−0.29 to 1.64
Normalized annual ∆SER (D)	−0.29 ± 0.28***	0.29 to −1.31
Normalized annual ∆AL (mm)	0.17 ± 0.13***	−0.13 to 0.62

***p < 0.001, unpaired *t*-test, comparing to control group (Annual ∆SER: −0.55 ± 0.36D and annual ∆AL: 0.32 ± 0.18 mm) and in the previous two-year DIMS randomized controlled trial (7).

### 3.3 Change in axial length in patients with previous myopic control intervention and combination intervention

Among 63 patients with history of myopia control, 14 of them switched to use DIMS lenses alone and 49 of them wore DIMS lenses together with using low dose atropine. The history of myopia control included low dose atropine (*n* = 1, duration of treatment = 5 months), orthokeratology (*n* = 3, duration treatment from 1 to 20 months), soft contact lenses (*n* = 1, duration of treatment = 3 months), progressive addition lenses (*n* = 2, duration of treatment = 12 months) and other specialized spectacle lenses for myopia control (*n* = 7, duration of treatment from 11 to 20 months). Table 3 shows the baseline demographics and ∆SER and ∆AL after DIMS lens wear. Patients (*n* = 426) with no myopia control experience were selected and compared with these 63 patients. Both groups had similar baseline age, SER, AL and wearing period. Those with myopia control history were slightly younger than those without myopic control although it did not reach significance (Myopic control vs. No myopic control; 8.25 ± 2.31 years vs. 8.82 ± 2.02 years, *p* = 0.109).

**Table 3:** Comparison of the baseline demographics and changes in myopia progression in terms of spherical equivalent refraction (SER) and axial length (AL) in patients with and without myopic control history.

Baseline	Myopia control history (*n* = 63)		No myopia control history (*n* = 426)		Mann–Whitney U test
	Mean ± SD	Range	Mean ± SD	Range	
Age (years)	8.25 ± 2.31	3 to 12	8.82 ± 2.02	5 to 17	*p* = 0.109
Male: female	39:24		202:224		*p* = 0.044^a^
Spherical equivalent refraction (D)	−2.88 ± 1.57	−0.75 to −8.00	−2.63 ± 1.27	−0.25 to −7.00	*p* = 0.202
Axial length (mm)	24.47 ± 0.92	22.64 to 27.35	24.62 ± 0.93	22.40 to 28.04	*p* = 0.615^b^
Wearing period (months)	32.33 ± 9.16	11.50 to 50.50	31.93 ± 10.10	11.50 to 48.10	*p* = 0.999
After wearing DIMS lenses					
Total ∆SER (D)	−1.17 ± 0.76	0.13 to −3.00	−0.91 ± 0.79	0.88 to −3.13	*p* = 0.008
Total ∆AL (mm)	0.66 ± 0.39	−0.03 to 1.60	0.54 ± 0.40	−0.29 to 1.72	*p* = 0.872^b^
Normalized annual ∆SER (D)	−0.46 ± 0.33	0.04 to −1.30	−0.36 ± 0.32	0.29 to −1.63	*p* = 0.027
Normalized annual ∆AL (mm)	0.25 ± 0.14	−0.01 to 0.59	0.21 ± 0.16	−0.13 to 0.78	*p* = 0.547^b^

^aChi-square test was used. bUnpaired t-test was used.^

It revealed that patients with myopia control history had greater myopia progression after wearing DIMS lenses than those without (Table 3). The total myopia progression in terms of ∆SER was significantly faster in the former group than the latter group (Total ∆SER in 32 months: −1.17 ± 0.76D vs. −0.91 ± 0.78D, *p* < 0.01; normalized annual ∆SER: −0.46 ± 0.33D vs. – 0.36 ± 0.32D, *p* < 0.05). Although the patients with myopia control history had a greater axial elongation compared to those with no prior myopia control history, it did not reach significance that could be due to the smaller sample size in patients with myopia control history (*p* = 0.872, comparing the total ∆AL between two groups).

### 3.4 Annual changes in axial length and other parameters

**Figure 2:**
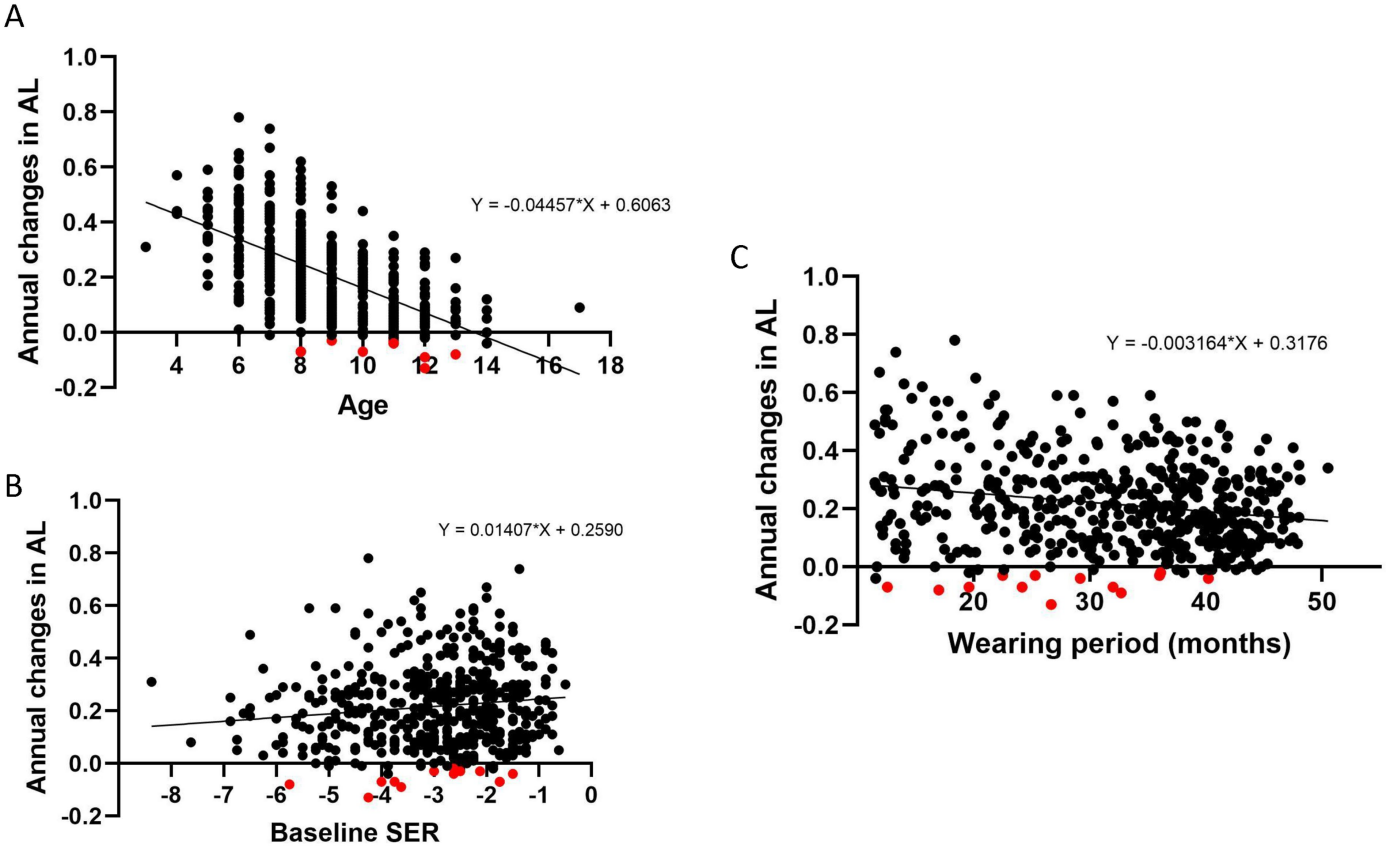
The relationship between annual changes in axial length (AL) and baseline age **(A)**, baseline spherical equivalent refraction (SER) **(B)**, and wearing period **(C)**. Both age and wearing period demonstrated a significant negative association with annual changes in axial length. Patients with shortening of axial length were indicated in red.

The relationship between the annual ∆AL and the baseline parameters such as age, baseline SER and wearing period were investigated. There was a significant negative correlation between age at baseline and annual ∆AL (correlation coefficient, *r* = −0.61, *p* < 0.001). Patients started wearing DIMS lenses at a young age had a greater annual ∆AL (Figure 2A). Although patients with mild baseline myopia had a great annual change in axial length (correlation coefficient, *r* = 0.14, *p* = 0.002, Figure 2B), the association was weak. There was also a weak negative association between the duration of wearing period and annual ∆AL (correlation coefficient, *r* = 0.16, *p* < 0.001, Figure 2C).

### 3.5 Shortening of axial length after DIMS lens wear

There were 13 patients (2.7%) with axial length shortening more than 0.05 mm after DIMS lens wear. Average duration of wearing period was 27.74 ± 8.13 months (range: 12.60 to 40.20 months, Table 4). Patients had a mean axial length shortening of 0.13 mm with the recession of myopia of 0.09D during the wearing period. All patients had no tropia and received DIMS lenses only as myopic control intervention. There were no significant associations between AL shortening and age, baseline SER and wearing period (Figure 1).

**Table 4:** Baseline demographics of patients who had shortening of axial length (*n* = 13) and comparison to those with no axial shortening (*n* = 476).

	Shortening of axial length (*n* = 13)		No shortening of axial length (*n* = 476)		Mann–Whitney U test
	Mean ± SD	Range	Mean ± SD	Range	
Age (years)	10.38 ± 1.66	8 to 13	8.70 2.06	3 to 17	*p* = 0.003
Male: female	6:7		242:234		*p* = 0.958^a^
Cycloplegic refraction	Yes: No = 10:3		Yes: No = 238:238		*p* = 0.102^a^
Baseline spherical refraction (D)	−2.65 ± 1.14	−5.50 to −1.50	−2.66 ± 1.31	−8.00 to −0.25	*p* = 0.942
Baseline cylindrical refraction (D)	−0.73 ± 0.62	−1.75 to 0.00	−0.73 ± 0.75	−4.00 to 0.00	*p* = 0.749
Baseline spherical equivalent refraction (D)	−3.02 ± 1.22	−5.75 to −1.50	−3.03 ± 1.39	−8.38 to −0.50	*p* = 0.944
Wearing period (months)	27.24 ± 8.13	12.60 to 40.20	32.11 ± 9.99	11.50 to 50.50	*p* = 0.046
Axial length (mm)	24.66 ± 0.80	23.34 to 25.69	24.60 ± 0.93	22.40 to 28.04	*p* = 0.691^b^
Total ∆SER (D)	0.09 ± 0.29	−0.50 to 0.50	−0.97 ± 0.78	−3.13 to 0.88	*p* < 0.001
Total ∆AL (mm)	−0.13 ± 0.07	−0.29 to −0.06	0.57 ± 0.39	−0.05 to 1.72	*p* < 0.001^b^
Normalized annual ∆SER (D)	0.03 ± 0.12	−0.18 to 0.23	−0.39 ± 0.32	−1.63 to 0.29	*p* < 0.001
Normalized annual ∆AL (mm)	−0.06 ± 0.03	−0.13 to −0.02	0.22 ± 0.15	−0.04 to 0.78	*p* < 0.001^b^

^aChi-square test was used. bUnpaired t-test was used.^

## 4 Discussion

The current study demonstrated the effect of DIMS lenses on axial elongation from a retrospective real-world study. One may argue that the axial length collected in the current study were come from two optical biometers. Previous studies showed that the repeatability of IOL Master 500 and AL-Scan was 0.05 mm (14, 15). Used either IOLMaster or AL-Scan, the 95% limits of agreement between these two instruments in axial length were within 0.05 mm (16–18). Therefore, the results shown in the current study were reliable.

Comparing to our previous DIMS randomized controlled study (7), the myopia progression in terms of axial elongation and SER was faster in the current real-world dataset (normalized annual ∆AL; current study vs. DIMS study (7); 0.22 ± 0.16 mm vs. 0.11 ± 0.18 mm). One of the underlying reasons could be due to the inclusion criteria of the current study. Besides, the presence of selection bias in the current retrospective study could attribute to the discrepancy. Various confounders such as the time spent at near work and outdoor activities, the parental myopia status could be the possible reasons.

We did not exclude the patients with prior myopia control interventions. This group of subjects particularly those with previous myopia control history were fast progressor in myopia, thus they have undergone various interventions before prescription of DIMS lenses. Axial elongation was more pronounced in the group with prior treatment compared to those without. This discrepancy may be attributed to a rebound effect following intervention changes. Additionally, age differences could have contributed, as patients with prior myopic control were slightly younger than those without such interventions. A larger sample size is necessary to accurately determine the underlying cause of this discrepancy. The current study also covered the COVID-19 pandemic period which increased myopia progression by 2.5-fold compared with pre-COVID-19 period (19) due to the increased screen time and less outdoor exposure. Therefore, our findings revealed that myopia progression was faster than the previous DIMS study performed in pre-COVID-19 period. More lockdown period also increased myopia progression and axial elongation, such negative impact affected single vision lenses wearers more (20).

Non-adherence to intervention plays a major role in discrepancy of treatment efficacy obtained from real-world data compared with randomized controlled trials. The reasons for non-adherence can be multifactorial and beyond our control, e.g., burden of periodic follow-up visits. This problem might not be the reason in myopia control using spectacles unless patients do not wear the spectacles long enough. Lam et al. (21) found that myopia progression was less in children wearing myopia control contact lenses for longer hours.

In the current study, both age and wearing period demonstrated a significant negative association with annual change in AL. The negative association between age and annual AL elongation was in line with the other studies, suggesting that myopia progression was faster in the children with younger age of onset (22, 23). Annual axial elongation was weakly associated with the wearing period of DIMS lens wear. It implied the effect of DIMS lenses on retarding axial elongation could be sustained with the increased duration of lens wear with a minimum decay effect. The adherence to DIMS lens wear might also account for the weak correlation, though this is considered unlikely due to the typically high compliance associated with spectacle lens use compared to other interventions. Sample size could be further increased to confirm the weak correlation in the further study.

We also observed a clinically significant shortening of axial length in children after wearing DIMS lenses for 2 years. A smaller proportion of patients (2.5%) experienced the axial shortening when compared to those myopic children after repeated low-level red light therapy (LLLT) for a year (24). Around 27% of children had shorter AL of at least 0.05 mm after LLLT, in which nearly 5% of them had more than 0.20 mm shortening of AL (25). The discrepancy could be due to different mechanism of defocus and red light in modulating eye growth. The exact mechanism in modulating the axial shortening by LLLT is largely unknown (26).

The magnitude of axial shortening in DIMS wearers was larger than those children after orthokeratology (DIMS vs. orthokeratology; −0.13 ± 0.07 mm vs. −0.08 ± 0.04 mm) (27). Also, the shortening of AL was sustained over 2 years of DIMS lenses while those under after orthokeratology only had the axial shortening for the first 7 months (27). However, increasing the sample size could better establish the association between AL shortening and DIMS lens wear. An increase in choroidal thickness could account for the axial shortening after the myopic control interventions. Choroidal thickness was found to increase significantly after exposure of various myopic control interventions such as orthokeratology (28), DIMS (29), atropine (30) and LLLT (31), although the magnitude and time course varied between interventions. The exact reason of axial shortening should be further investigated because choroidal thickness was not measured in the current study.

The study has certain limitations that should be acknowledged. Our analysis did not include patients wearing single vision spectacle lenses, which prevented us from calculating and comparing the effectiveness of Defocus Incorporated Multiple Segments (DIMS) lenses for myopia control with those wearing single vision lenses as a control group. It was anticipated that the effectiveness of DIMS lenses would be lower compared to previous randomized controlled trials. This discrepancy could be attributed to parental decisions and the myopia progression profile of the children. In real-world settings, children wearing single vision lenses typically exhibit stable myopia progression, leading their parents to choose to continue using single vision lenses.

Another limitation of the study was the relatively small sample size when compared to other real-world studies. This may limit the generalizability of the current results. To gain more insight into a broader population, it would be valuable to increase the dataset by including participants from different geographical locations. Additionally, individuals over the age of 18 who were wearing DIMS lenses were excluded from the study. However, it is important to note that axial elongation can still occur in adults with high myopia (32). Consequently, the findings of this study are limited to youngsters and children. These limitations should be considered when interpreting the results, and further research with larger sample sizes and inclusion of different age groups is warranted to gain a more comprehensive understanding of the effects of DIMS lenses on myopia control.

## Data Availability

The raw data supporting the conclusions of this article will be made available by the authors, without undue reservation.
